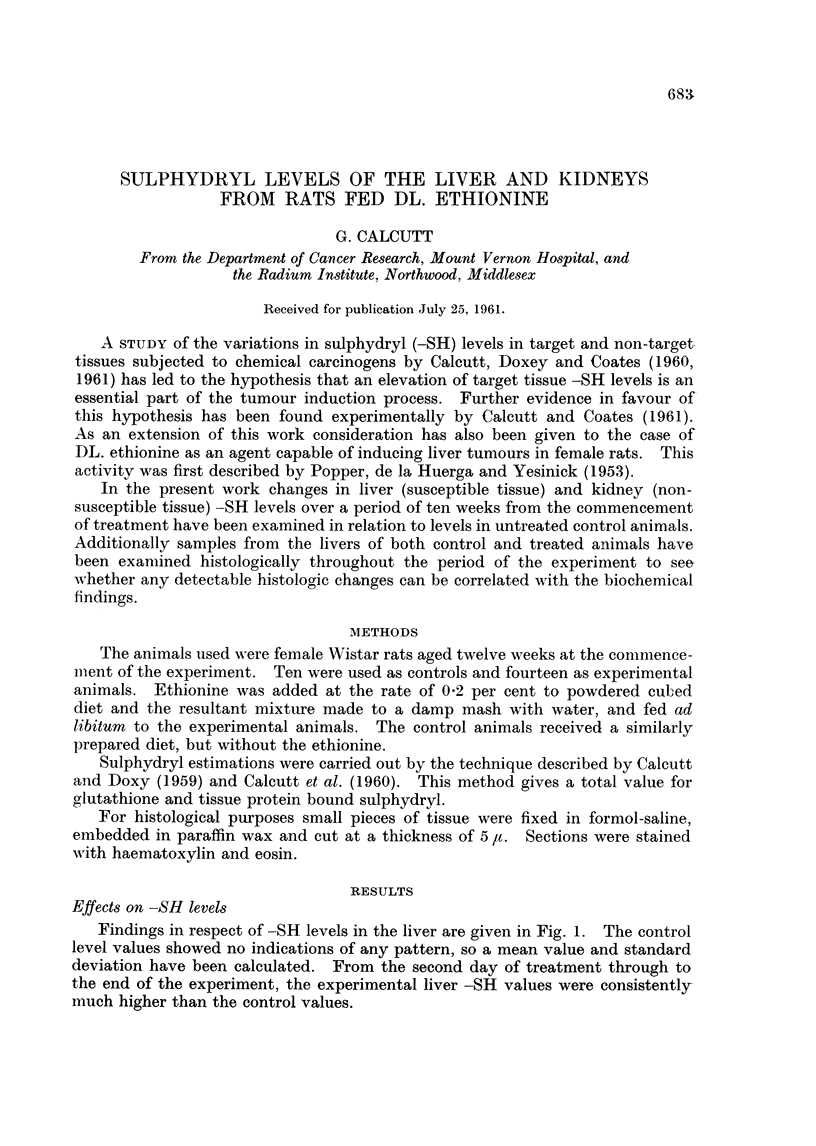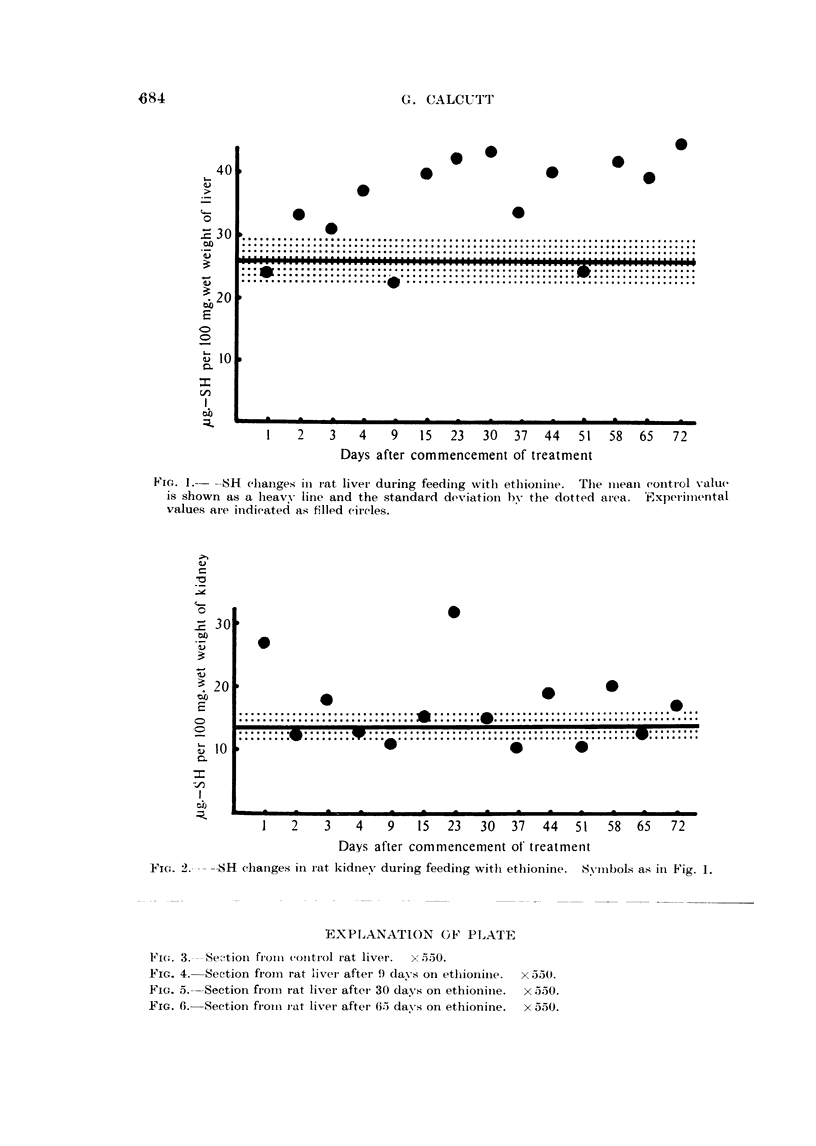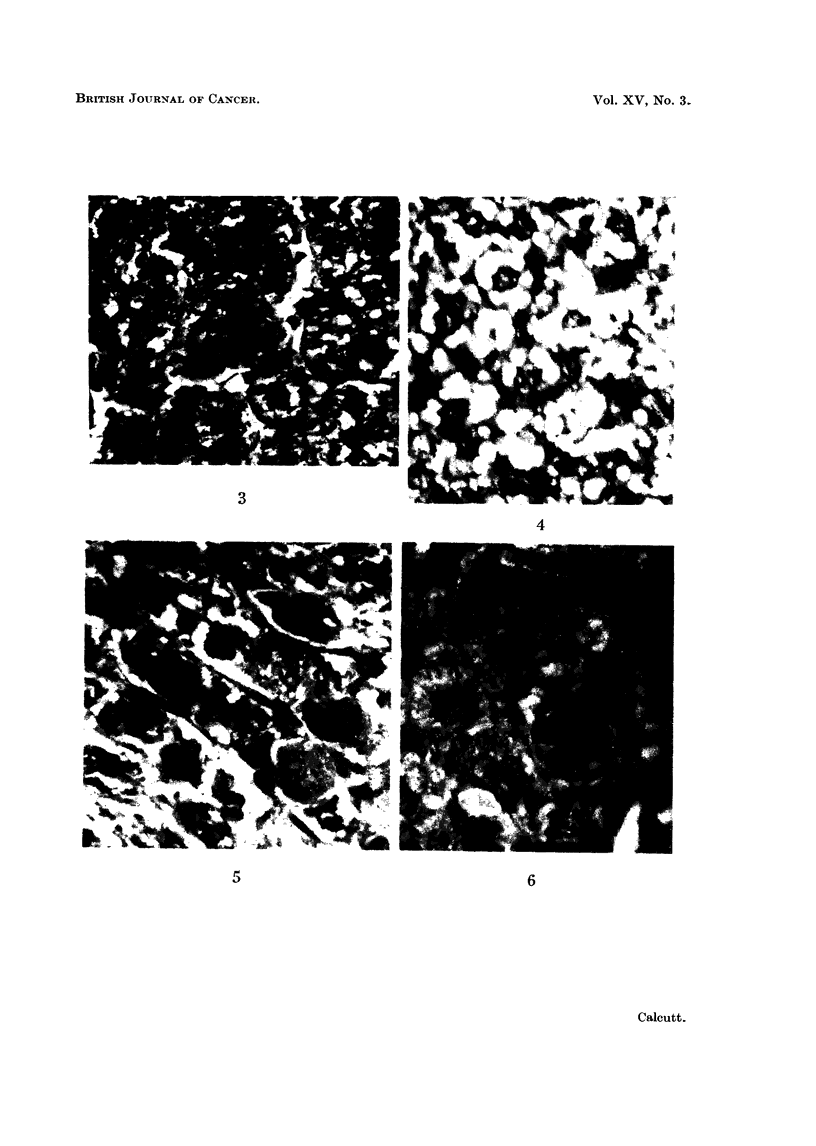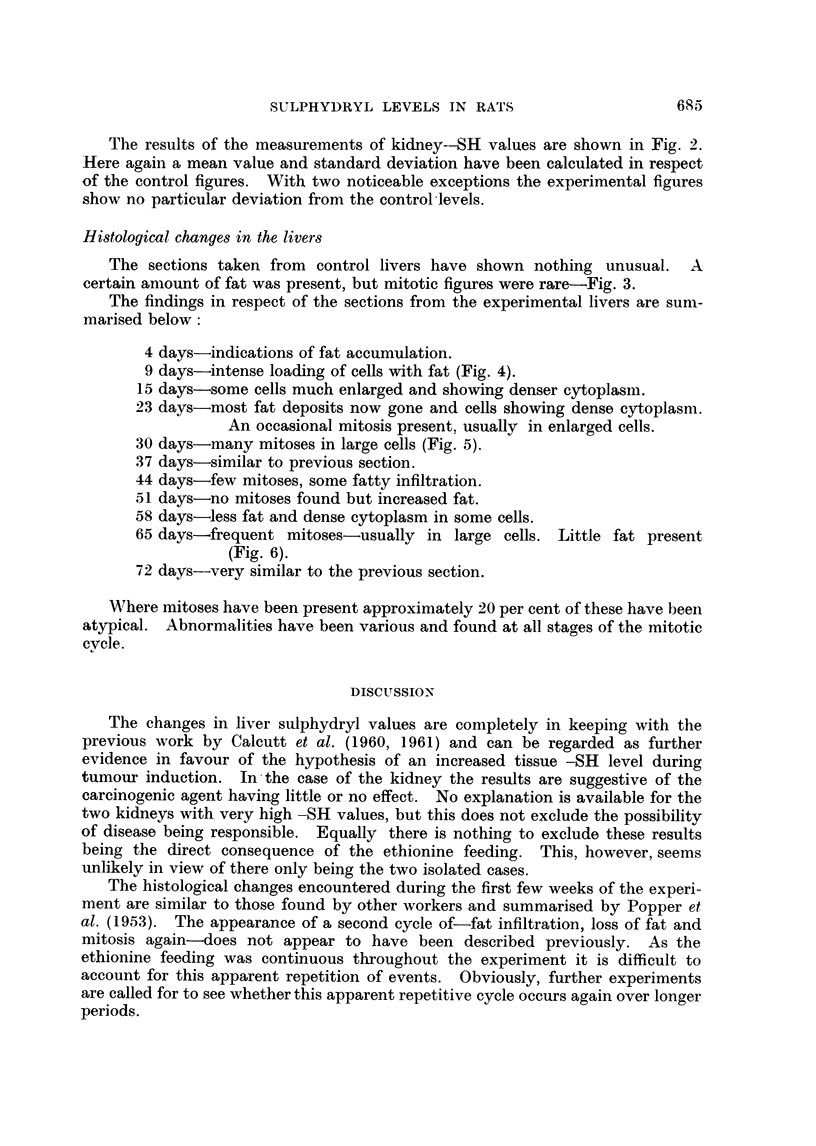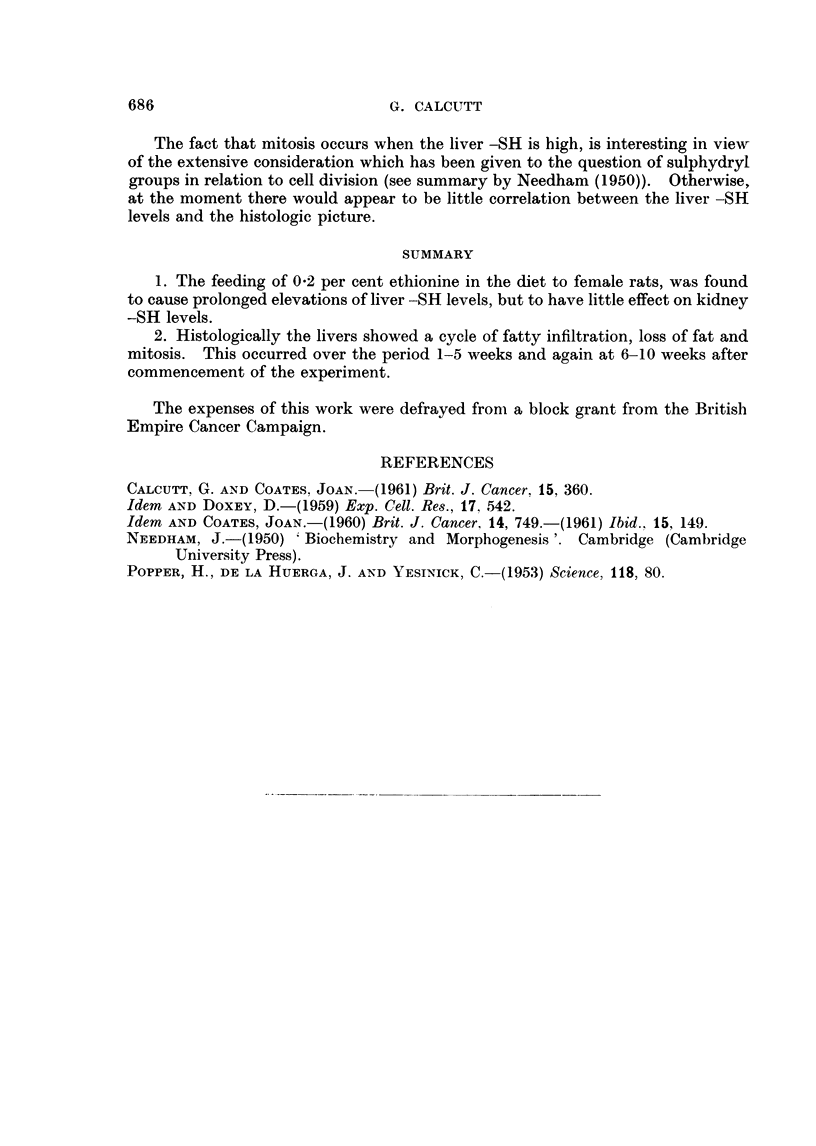# Sulphydryl Levels of the Liver and Kidneys from Rats Fed DL. Ethionine

**DOI:** 10.1038/bjc.1961.77

**Published:** 1961-09

**Authors:** G. Calcutt

## Abstract

**Images:**


### SULPHYDRYL LEVELS OF THE LIVER AND KIDNEYS FROM RATS FED DL. ETHIONINE

G. CALCUTT

*From the Department of Cancer Research, Mount Vernon Hospital, and the Radium Institute, Northwood, Middlesex*

Received for publication July 25, 1961.

A STUDY of the variations in sulphydryl (–SH) levels in target and non-target tissues subjected to chemical carcinogens by Calcutt, Doxey and Coates (1960, 1961) has led to the hypothesis that an elevation of target tissue –SH levels is an essential part of the tumour induction process. Further evidence in favour of this hypothesis has been found experimentally by Calcutt and Coates (1961). As an extension of this work consideration has also been given to the case of DL. ethionine as an agent capable of inducing liver tumours in female rats. This activity was first described by Popper, de la Huerga and Yesinick (1953).

In the present work changes in liver (susceptible tissue) and kidney (non-susceptible tissue) –SH levels over a period of ten weeks from the commencement of treatment have been examined in relation to levels in untreated control animals. Additionally samples from the livers of both control and treated animals have been examined histologically throughout the period of the experiment to see whether any detectable histologic changes can be correlated with the biochemical findings.

### METHODS

The animals used were female Wistar rats aged twelve weeks at the commencement of the experiment. Ten were used as controls and fourteen as experimental animals. Ethionine was added at the rate of 0·2 per cent to powdered cubed diet and the resultant mixture made to a damp mash with water, and fed *ad libitum* to the experimental animals. The control animals received a similarly prepared diet, but without the ethionine.

Sulphydryl estimations were carried out by the technique described by Calcutt and Doxy (1959) and Calcutt *et al.* (1960). This method gives a total value for glutathione and tissue protein bound sulphydryl.

For histological purposes small pieces of tissue were fixed in formol-saline, embedded in paraffin wax and cut at a thickness of 5 $\mu$. Sections were stained with haematoxylin and eosin.

### RESULTS

### *Effects on –SH levels*

Findings in respect of –SH levels in the liver are given in Fig. 1. The control level values showed no indications of any pattern, so a mean value and standard deviation have been calculated. From the second day of treatment through to the end of the experiment, the experimental liver –SH values were consistently much higher than the control values.

FIG. 1.— -SH changes in rat liver during feeding with ethionine. The mean control value is shown as a heavy line and the standard deviation by the dotted area. Experimental values are indicated as filled circles.

FIG. 2.— -SH changes in rat kidney during feeding with ethionine. Symbols as in Fig. 1.

### EXPLANATION OF PLATE

FIG. 3.—Section from control rat liver. ×550.

FIG. 4.—Section from rat liver after 9 days on ethionine. ×550.

FIG. 5.—Section from rat liver after 30 days on ethionine. ×550.

FIG. 6.—Section from rat liver after 65 days on ethionine. ×550.

3

4

5

6

The results of the measurements of kidney–SH values are shown in Fig. 2. Here again a mean value and standard deviation have been calculated in respect of the control figures. With two noticeable exceptions the experimental figures show no particular deviation from the control levels.

*Histological changes in the livers*

The sections taken from control livers have shown nothing unusual. A certain amount of fat was present, but mitotic figures were rare—Fig. 3.

The findings in respect of the sections from the experimental livers are summarised below :

4 days—indications of fat accumulation.
9 days—intense loading of cells with fat (Fig. 4).
15 days—some cells much enlarged and showing denser cytoplasm.
23 days—most fat deposits now gone and cells showing dense cytoplasm. An occasional mitosis present, usually in enlarged cells.
30 days—many mitoses in large cells (Fig. 5).
37 days—similar to previous section.
44 days—few mitoses, some fatty infiltration.
51 days—no mitoses found but increased fat.
58 days—less fat and dense cytoplasm in some cells.
65 days—frequent mitoses—usually in large cells. Little fat present (Fig. 6).
72 days—very similar to the previous section.

Where mitoses have been present approximately 20 per cent of these have been atypical. Abnormalities have been various and found at all stages of the mitotic cycle.

### DISCUSSION

The changes in liver sulphydryl values are completely in keeping with the previous work by Calcutt *et al.* (1960, 1961) and can be regarded as further evidence in favour of the hypothesis of an increased tissue –SH level during tumour induction. In the case of the kidney the results are suggestive of the carcinogenic agent having little or no effect. No explanation is available for the two kidneys with very high –SH values, but this does not exclude the possibility of disease being responsible. Equally there is nothing to exclude these results being the direct consequence of the ethionine feeding. This, however, seems unlikely in view of there only being the two isolated cases.

The histological changes encountered during the first few weeks of the experiment are similar to those found by other workers and summarised by Popper *et al.* (1953). The appearance of a second cycle of—fat infiltration, loss of fat and mitosis again—does not appear to have been described previously. As the ethionine feeding was continuous throughout the experiment it is difficult to account for this apparent repetition of events. Obviously, further experiments are called for to see whether this apparent repetitive cycle occurs again over longer periods.

The fact that mitosis occurs when the liver –SH is high, is interesting in view of the extensive consideration which has been given to the question of sulphydryl groups in relation to cell division (see summary by Needham (1950)). Otherwise, at the moment there would appear to be little correlation between the liver –SH levels and the histologic picture.

SUMMARY

1. The feeding of 0·2 per cent ethionine in the diet to female rats, was found to cause prolonged elevations of liver –SH levels, but to have little effect on kidney –SH levels.

2. Histologically the livers showed a cycle of fatty infiltration, loss of fat and mitosis. This occurred over the period 1–5 weeks and again at 6–10 weeks after commencement of the experiment.

The expenses of this work were defrayed from a block grant from the British Empire Cancer Campaign.

REFERENCES

CALCUTT, G. AND COATES, JOAN.—(1961) *Brit. J. Cancer*, **15**, 360.
*Idem* AND DOXEY, D.—(1959) *Exp. Cell. Res.*, **17**, 542.
*Idem* AND COATES, JOAN.—(1960) *Brit. J. Cancer*, **14**, 749.—(1961) *Ibid.*, **15**, 149.
NEEDHAM, J.—(1950) ' Biochemistry and Morphogenesis '. Cambridge (Cambridge University Press).
POPPER, H., DE LA HUERGA, J. AND YESINICK, C.—(1953) *Science*, **118**, 80.